# Efficiency of noninvasive prenatal testing for the detection of fetal microdeletions and microduplications in autosomal chromosomes

**DOI:** 10.1002/mgg3.1339

**Published:** 2020-06-15

**Authors:** Yuanyuan Pei, Liang Hu, Jinxing Liu, Lijuan Wen, Xiaojin Luo, Jian Lu, Fengxiang Wei

**Affiliations:** ^1^ Genetic Laboratory of Shenzhen Longgang Maternal and Child Health Hospital Shenzhen Guangdong China; ^2^ Medical Genetic Centre of Guangdong Women and Children Hospital Guangzhou Guangdong China; ^3^ Department of Cell Biology Jiamusi University Jiamusi Heilongjiang China; ^4^ Department of Pathogenic Microorganisms Zunyi Medical University Zunyi Guangdong China; ^5^ School of Public Health Anhui Medical University Hefei Anhui China

**Keywords:** chromosomal microarray, copy number variation, karyotype analysis, noninvasive prenatal testing, positive predictive value

## Abstract

**Background:**

Noninvasive prenatal testing (NIPT) is commonly used to screen for fetal genetic abnormalities. However, the ability of NIPT to detect copy number variations (CNVs) has not been reported. Accordingly, in this study, we analyzed the efficiency of NIPT for the detection of fetal autosomal CNVs.

**Methods:**

Patients who were positive for autosomal CNVs by NIPT and underwent diagnostic studies by karyotype analysis and chromosomal microarray (CMA) were evaluated. Samples were divided into groups according to age, in vitro fertilization, fetal‐free DNA concentration, uniquely mapped reads number, CNV size, and CNV type.

**Results:**

Chromosomal microarray showed that the positive predictive value (PPV) of autosomal CNVs detected by NIPT was 14.89%. Increasing fetal DNA concentrations and uniquely mapped read numbers did not affect the PPV of CNVs detected by NIPT. There were no differences between microduplication and microdeletion PPVs detected by NIPT. The PPV of CNVs less than 10 Mb was significantly higher than that of CNVs greater than 10 Mb detected by NIPT.

**Conclusion:**

The accuracy of NIPT for autosomal CNVs needs to be improved.

## Graphical abstract

The positive predictive value of copy number variations (CNVs) less than 10 Mb was significantly higher than that of CNVs greater than 10 Mb detected by Noninvasive prenatal testing (NIPT). NIPT was effective for detecting CNVs less than 10 Mb.

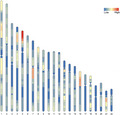

## 1 INTRODUCTION

Copy number variations (CNVs) are important factors affecting human phenotypic variations and diseases (Sebat et al., 2004). Some CNVs can cause fetal microdeletion or microduplication (MD) syndromes that are not related to the age of the pregnant mother (Miller et al., 2010; Wapner et al., 2015). Therefore, the risk of MD in offspring is probably higher than that of Down's syndrome in young pregnant women. Several recent studies have shown that the incidence of MD syndrome in fetuses with normal maternal chromosomes is 1%–1.7% (Wapner et al., 2012). Williams syndrome, DiGeorge/velo‐cardio‐facial syndrome, and Prader–Willi syndrome are the most common MD syndromes encountered in clinical practice (Cheung et al., 2005). Moreover, approximately 12% of unexplained mental retardation, multiple malformations, and stunting are caused by MD syndromes (Miller et al., 2010). Therefore, small chromosome‐imbalanced aberrations should be screened for during genetic consultations and prenatal diagnoses.

In parallel with high‐throughput sequencing, cell‐free DNA‐based noninvasive prenatal testing (NIPT) for fetal aneuploidies is rapidly becoming a first‐tier screening test in clinical practice (Chen et al., 2019). This approach has been validated in multiple clinical cohorts, demonstrating that NIPT is highly sensitive and specific for patients at increased risk of T13, T18, and T21 aneuploidies, which are the main pathogeneses of chromosomal disorders in neonates (Gil, Accurti, Santacruz, Plana, & Nicolaides, 2017). Additionally, low‐depth whole‐genome sequencing results of NIPT can also be indicated for other chromosomal abnormalities, including aneuploidies in other chromosomes and CNVs (Chen et al., 2019; Deng et al., 2019; Pös et al., 2019).

Since the first application of NIPT in the prenatal screening of fetal CNVs reported in 2011 (Peters et al., 2011), many institutions have studied the utility of using NIPT to detect CNVs (Pös et al., 2019; Zhu et al., 2019). However, because of the placental origin of fetal DNA owing to the low sequencing depth or low production of fetal DNA template, NIPT yields false‐negative and false‐positive results during the screening of CNVs (Xu et al., 2016; Yin et al., 2015). Accordingly, the development of prenatal diagnostic approaches that do not induce adverse fetal outcomes is urgently required.

Karyotype analysis is a classical, commonly used approach in prenatal diagnosis. However, this approach has obvious limitations in the detection of CNVs owing to its low resolution. Chromosomal microarray (CMA) is a high‐resolution whole‐genome screening technology that can be used to analyze the presence of CNVs (Hay et al., 2018). Accordingly, in this study, we performed a retrospective statistical analysis to evaluate the efficiency of NIPT for detecting autosomal CNVs by analyzing NIPT, karyotype, and CMA results.

## 2 MATERIAL AND METHODS

### 2.1 Ethical compliance

This project was approved by the ethics committee of Shenzhen Longgang Maternal and Child Health Hospital, and informed consent was obtained from all patients.

### 2.2 Study populations

This study evaluated 36,599 pregnant women who received prenatal care at Shenzhen Longgang Maternal and Child Health Hospital from December 2017 to June 2019. CNVs were detected in 330 participants by NIPT and 141 of these patients then underwent prenatal diagnosis using chromosomal karyotyping and CMA. Based on the karyotype and CMA results, we analyzed the accuracy of NIPT for the detection of autosomal CNVs.

### 2.3 NIPT

For each patient, 5 ml of venous blood was collected using EDTA‐K_2_ tubes and centrifuged at 4°C and 1,600 × *g* for 10 min within 8 hr after blood collection. The plasma was then centrifuged at 4°C and 16,000 × *g* for 10 min to obtain cell‐free plasma, which was stored at −80°C. According to the standard operating procedures (BGI), cell‐free DNA was extracted from maternal plasma, and DNA libraries were constructed using an enzyme reaction, molecular labels, and polymerase chain reaction amplification. The DNA nanospheres were generated by rolling ring replication and loaded into the sequencing chip. Each sample was sequenced using a BGIseq‐500 platform with the combined probe anchored polymer sequencing method, and the BGI Halos software was used to perform the bioinformatic analysis. The quality control parameters were as follows: library concentration was higher than 4 ng/μL; unique mapped reads number was higher than 6 × 10^6^; GC content was 38%–42%; and fetal DNA fraction was higher than 3.5%.

### 2.4 Karyotype analysis

For karyotype analysis, 18–20 ml of amniotic fluid was obtained by amniocentesis. The amniotic fluid cells were transferred to 25‐cm^2^ culture bottles with 4.5 ml medium (He Neng Bio, China) and cultured in a 37°C incubator with 5% carbon dioxide. On Day 7, the culture medium was replaced if more than five cell colonies were observed. The cells were harvested at 10–12 days if more than 10 cell colonies were observed. After colchicine treatment for 2 hr, cells were digested using 1:250 trypsin (HyClone), and incubated with 0.075 M KCl for 30 min. Following the procedure for prefixation, fixation, dropping, baking, and G‐band staining, metaphases with more than 320–400 G‐bands were accepted for karyotype analysis. Karyotypes were scanned and analyzed on a Leica GL120 system. The naming and specification of abnormal karyotypes were based on ISCN 2016.

### 2.5 CMA

Affymetrix CytoScan 750k chips were used for CMA analysis following CytoScan Assays according to local experimental standards. DNA extraction (Qiagen), hybridization, and whole‐genome scanning were performed, and data analysis was carried out using Affymetrix Chromosome Analysis Suite Software (version 3.1.0.15). Only unbalanced rearrangements (structural losses and duplications) were considered for chromosome abnormalities; balanced translocations, inversions, and loss of heterozygosity were not included. CNVs were classified and verified through OMIM, UCSC, International Standard Cytogenomic Array (ISCA), Database of Genome Variants (DGV), and Decipher databases and divided into five categories (pathogenic, likely pathogenic, uncertain significance, likely benign, and benign). For CNVs of uncertain significance, CMA was further performed on parents to verify whether the CNVs were inherited from parents with a normal phenotype. CNVs inherited from parents with a normal phenotype were regarded as benign.

### 2.6 Statistics

Excel and R language were used for data statistical analysis. Continuous variables were presented as medians and interquartile ranges (IQRs), and categorical variables were presented as *n* (%). In total, 330 samples with abnormal autosomal structures indicated by NIPT were divided into groups according to age, in vitro fertilization (IVF), fetal‐free DNA concentration, uniquely mapped reads number, CNV size, and CNV type. The prenatal diagnosis rate for each group was calculated and the positive predictive values (PPVs) of CNVs detected by NIPT were calculated based on CMA results. Fisher exact probability tests were used for comparing CNV PPVs for NIPT among different groups. Results with *p* values of less than .05 were considered statistically significant.

## 3 RESULTS

### 3.1 Population profiles

**FIGURE 1:**
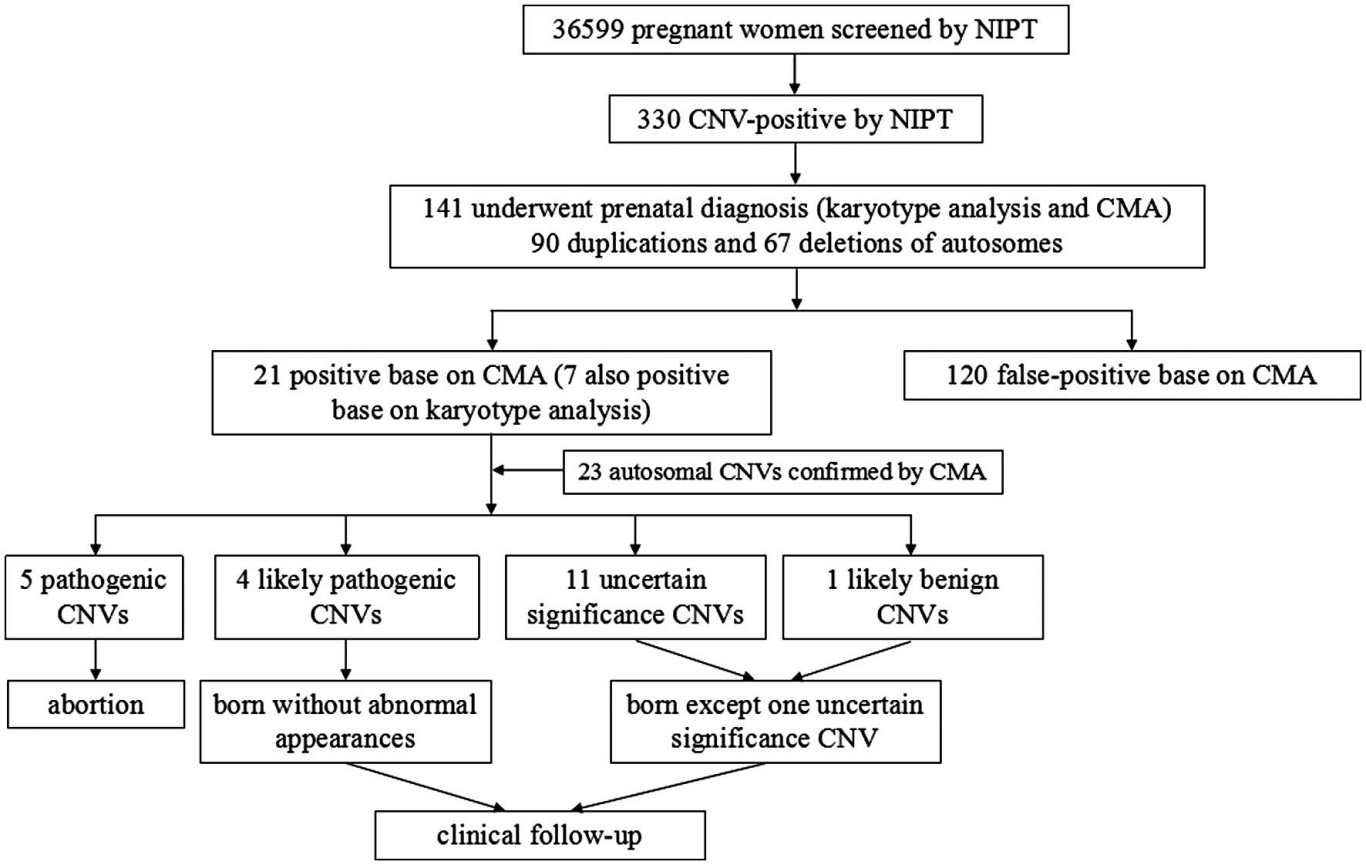
Enrollment, outcome classification, and follow‐up of the pregnant women participating in the NIPT, karyotype analysis, and CMA examinations. CMA, chromosomal microarray; NIPT, noninvasive prenatal testing

Enrollment, outcome classification, and follow‐up of the pregnant women participating in this study are presented in Figure 1. In total, 141 pregnant women with complete results for NIPT, karyotyping, and CMA were included in the study. The statistical profiles of maternal age, weight, gestational age (days), gravidity, parity, fetal‐free DNA concentration, uniquely mapped reads, and CNV size are shown in Table 1.

**TABLE 1:** Characteristics of study subjects

Clinical characteristics	Data
Samples	141
Age (years)	28 (26–32)
Maternal weight (kg)	54 (49.5–60.0)
Gestational age (days)	117 (97–123)
Gravidity [*N* (%)]	
<3	86 (60.56)
≥3	55 (39.44)
Parity [*N* (%)]	
0	55 (38.73)
1	73 (52.11)
>1	13 (9.16)
cffDNA^a^ concentration（%）	7.89 (6.18–10.76)
Uniquely mapped reads (M)	10.59 (9.06–11.87)
CNV^b^ size (Mb)	15.73 (7.09–18.87)
Duplications	13.87 (4.46–16.96)
Deletions	16.71 (10.37–23.11)

Abbreviation: CNV, copy number variation.

^a^ Fetal‐free DNA.

^b^ Copy number variation.

### 3.2 NIPT

**FIGURE 2:**
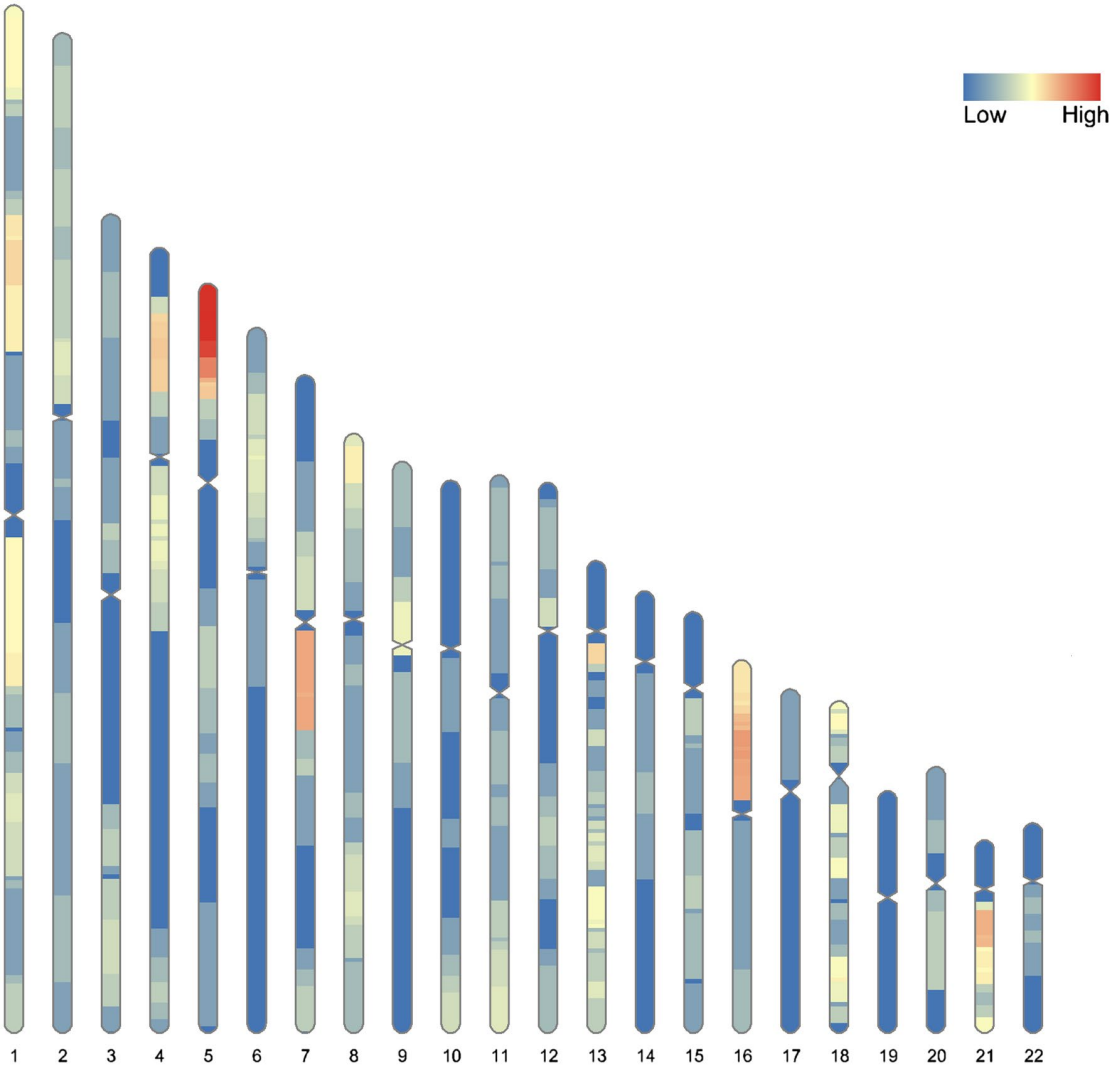
Autosomal CNVs (*n* = 157) detected by NIPT were distributed on each autosome, except chromosome 19, and CNVs on chromosomes 5, 7, and 16 were the most common. CNV, copy number variation; NIPT, noninvasive prenatal testing

Among the 141 specimens, 90 duplications and 67 deletions of autosomes were found, with a total of 157 CNVs. Among them, single deletion or single duplication was detected in 129 specimens, and more than two abnormalities were detected in 12 specimens. CNVs were distributed in each autosome, except chromosome 19, and CNVs on chromosomes 5, 7, and 16 were the most common, as shown in Table 2 and Figure 2.

**TABLE 2:** Autosomal CNVs detected by NIPT, karyotyping analysis, and CMA

Location	NIPT^a^	Karyotyping analysis	CMA^b^
chr1	9	0	0
chr2	2	0	0
chr3	4	0	0
chr4	12	1	3
chr5	29	1	3
chr6	3	0	0
chr7	18	1	1
chr8	5	1	1
chr9	3	0	0
chr10	2	1	1
chr11	4	1	2
chr12	6	0	1
chr13	9	0	1
chr14	1	0	0
chr15	4	0	0
chr16	17	0	3
chr17	1	0	0
chr18	15	0	4
chr19	0	0	0
chr20	2	0	0
chr21	9	1	3
chr22	2	0	0
Total	157	7	23

Abbreviations: CMA, chromosomal microarray; CNV, copy number variation; NIPT, noninvasive prenatal testing.

^a^ Noninvasive prenatal testing.

^b^ Chromosomal microarray.

### 3.3 Comparison of NIPT, karyotyping, and CMA results

Among the 141 samples with complete information, CNVs through CMA were observed in 21 samples; seven of these samples had abnormal karyotypes according to karyotyping analysis (Table 3).

**TABLE 3:** CNVs confirmed by karyotyping analysis and CMA

No.	NIPT^a^	karyotyping analysis	CMA^b^	Comments	Source
1	del(18q21.33‐q22.2,6.35M); dup(18q12.1‐q12.2,5.35M)	46 XN	arr[hg19] 18q12.1q12.2(29,007,572–33,493,574)x3	Likely pathogenic	Maternal
2	del(18q22.1‐q22.3,4.11M)	46 XN	arr[hg19] 18q22.1q22.3(66,637,301–69,388,484)x1	Uncertain significance	Unverified
3	del(21q11.2‐q22.11,18.28M)	45,XN,‐21,der(22)t(21;22)(q22.1;p11.2)	arr[hg19] 21q11.2q22.11(15,016,486–33,575,521)x1	Pathogenic	Unverified
4	del(4q13.1‐q13.3,10.43M)	46,XN,del(4)(q13.1q13.3)	arr[hg19] 4q13.1q13.3(63,970,999–73,525,454)x1	Pathogenic	Unverified
5	del(5p14.3‐p14.1,6.09M)	46 XN	arr[hg19] 5p14.3p14.1(21,278,478–26,440,483)x1	Uncertain significance	Maternal
6	del(5p15.31‐p13.2,27.37M)	46,XN,del(5)(p13)	arr[hg19] 5p15.33p13.2(113,576–34,828,016)x1	Pathogenic	Unverified
7	dup(10q11.21‐q21.1,9.97M)	46,XN,dup(10)(q11.21)	arr[hg19] 10q11.21q21.1(45,641,650–54,583,026)x3	Likely pathogenic	Unverified
8	dup(11p15.4‐p15.1,10.77M)	46,XN,dup(11)(p15.4p15.2)	arr[hg19] 11p15.4p15.2(5,496,603–15,822,328)x3	Pathogenic	Unverified
9	dup(11q22.3‐q23.1,8.89M)	46 XN	arr[hg19] 11q22.3q23.2(103,171,064–112,775,136)x3	Likely pathogenic	Maternal
10	dup(12p13.32‐p12.2,15.98M)	46 XN	arr[hg19] 12p13.33(173,786–1,962,452)x1	Pathogenic	Non‐genetic
11	dup(13q33.1‐q33.2,3.09M)	46 XN	arr[hg19] 13q33.1q33.2(103,459,820–105,576,766)x3	Uncertain significance	Maternal
12	dup(16p13.12‐p11.2,19.61M)	46 XN	arr[hg19] 16p13.11(15,499,445–16,289,059)x3	Uncertain significance	Unverified
13	dup(16q23.1‐q24.3,13.87M)	46,XN,del(7)(q11.2q21）	arr[hg19] 7q11.23q21.11(72,329,724–80,081,782)x1 16q23.1(76,409,614–77,461,469)x3	Uncertain significance	Unverified
14	dup(18p11.32‐p11.31,5.84M)	46 XN	arr[hg19] 18p11.32(136,227–1,154,109)x1	Uncertain significance	Unverified
15	dup(18q11.2‐q12.1,2.90M)	46 XN	arr[hg19] 16p13.11p12.3(15,338,152–18,172,468)x3 18q12.1(25,368,075–27,292,515)x3	Uncertain significance	Unverified
16	dup(21q22.3,2.79M)	46 XN	arr[hg19] 21q22.3(45,703,830–47,932,298)x3	Uncertain significance	Unverified
17	dup(21q22.3,3.47M)	46 XN	arr[hg19] 21q22.3(42,641,330–47,858,476)x3	Likely pathogenic	Unverified
18	dup(4q12‐q13.1,5.42M)	46 XN	arr[hg19] 4q12q13.1(58,193,591–62,730,657)x3	Uncertain significance	Unverified
19	dup(4q12‐q13.2,10.41M)	46 XN	arr[hg19] 4q12q13.1(58,193,591–62,730,657)x4	Likely benign	maternal
20	dup(5q22.3‐q23.2,7.09M)	46 XN	arr[hg19] 5q22.3q23.2(114,707,119–122,547,523)x3	Uncertain significance	maternal
21	dup(8q11.21‐q12.1,7.96M)	46,XN,dup(8)(p11.2p12）	arr[hg19] 8q11.1q11.22(46,839,735–52,386,593)x3	Uncertain significance	Unverified

Abbreviations: CMA, chromosomal microarray; CNV, copy number variation; NIPT, noninvasive prenatal testing.

^a^ Noninvasive prenatal testing.

^b^ Chromosomal microarray.

**FIGURE 3:**
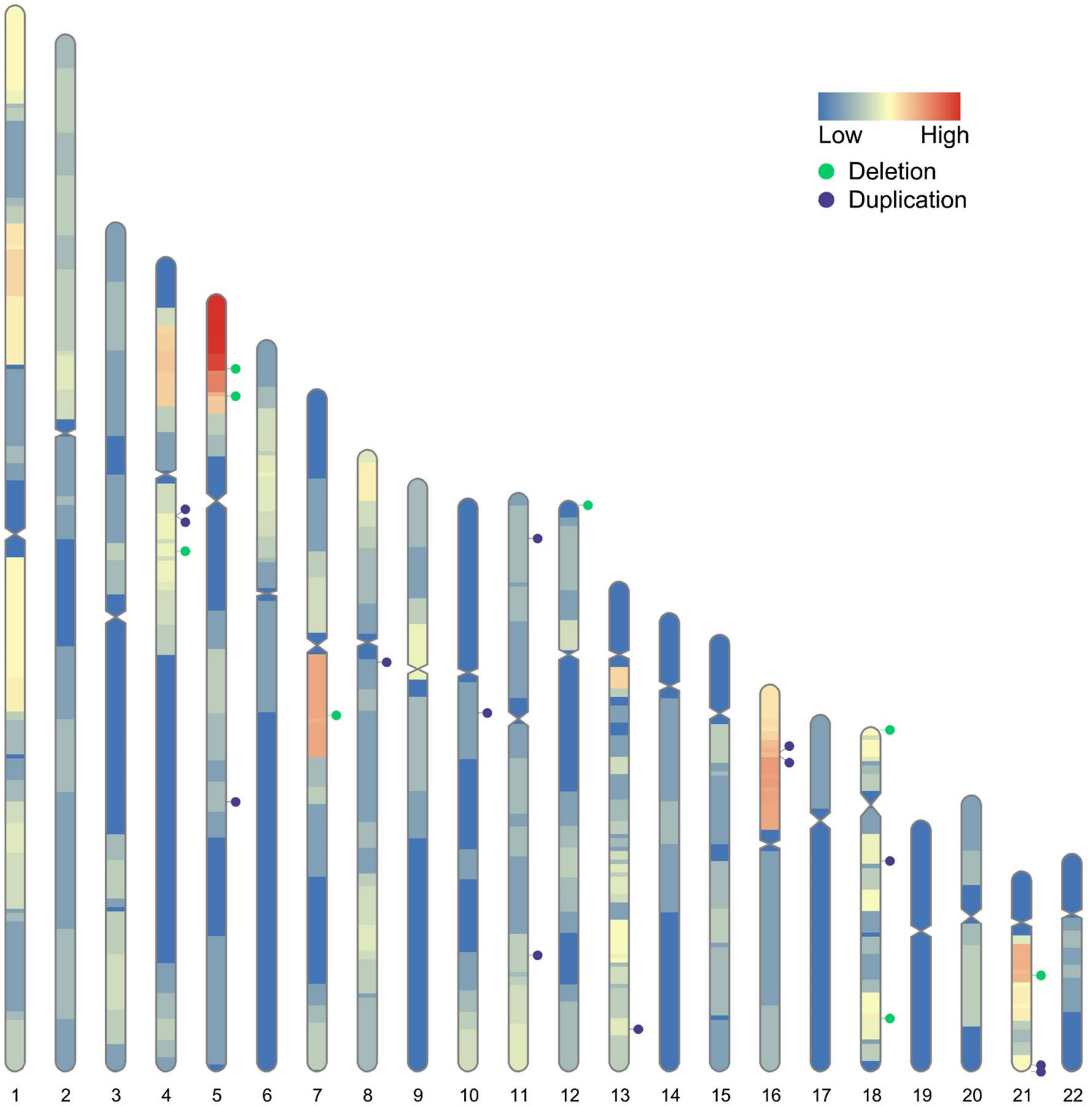
Distribution of autosomal 23 CNVs diagnosed by CMA. CMA, chromosomal microarray; CNV, copy number variation

Seven autosomal CNVs were detected by karyotyping analysis and six of the seven CNVs were detected by NIPT. In one sample, NIPT indicated a 13.87‐Mb duplication of the long arm of chromosome 16, whereas chromosome karyotype analysis indicated a partial deletion of the long arm of chromosome 7 (Table 3). Moreover, 23 autosomal CNVs were detected by CMA (Figure 3), 21 CNVs were confirmed in 21 NIPT samples, and the other two CNVs were the additional findings of CMA. Among the 23 CNVs detected by CMA, five were pathogenic, four were likely pathogenic, 13 were of uncertain significance, and one was likely benign.

In order to determine the sources of these CNVs, seven pregnant women and their spouses underwent CMA analysis of peripheral blood. Finally, two likely pathogenic CNVs, three CNVs of uncertain significance, one CNV with likely benign origin from the fetal mother, and one de novo pathogenic CNV were identified (Table 3).

In patient 1, NIPT showed a deletion of 6.35 Mb of the long arm of chromosome 18, with a 5.35‐Mb duplication. No abnormalities in the karyotype were detected and only a 4.49‐Mb duplication was detected in CMA. In patient 13, NIPT indicated a 13.87‐Mb long‐arm duplication of chromosome 16, karyotype analysis indicated a partial deletion of the long arm of chromosome 7, and CMA detected both chromosome 16 long‐arm duplications and chromosome 7 long‐arm deletions. In patient 15, NIPT showed a long‐arm duplication of chromosome 18, but short‐arm duplication of chromosome 16 was also detected in CMA.

### 3.4 Analysis of the efficiency of NIPT in CNV detection

Based on the CMA results, the false‐positive rate of NIPT for CNV was 85.11% (120/141). The PPV of CNV detected by NIPT was 14.89% (21/141).

Next, the PPVs of CNVs detected by NIPT in each group were calculated according to the maternal age, IVF, fetal‐free DNA concentration, uniquely mapped reads number, CNV size, and CNV type. The results showed that there was a significant difference in PPVs between CNVs of greater than or less than 10 Mb; the PPVs of CNVs less than 10 Mb detected by NIPT were higher than those of CNVs greater than 10 Mb (Table 4).

**TABLE 4:** Efficiency of NIPT for detecting CNVs at varies factors

Factors	Samples (*n*)	Prenatal diagnosed	Prenatal diagnosis rate (%)	True positive	PPV (%)	*p* *
Age						
≥35 years	43	12	27.91	1	8.33	1.00
<35 years	287	129	44.95	20	15.5	
IVF^a^						
Yes	11	8	72.73	1	12.5	1.00
No	319	133	41.69	20	15.04	
FF^b^						
≥10%	117	45	38.46	7	15.56	1.00
<10%	213	96	45.07	14	14.58	
Uniquely mapped reads (M)						
≥10	202	89	44.06	11	12.36	.35
<10	128	52	40.63	10	19.23	
CNV^c^ size						
≥20 Mb	70	32	45.71	1	3.13	1.674*E‐5
10−20 Mb	158	64	40.51	2	3.13	
<10 Mb	102	45	44.12	18	40.00	
Type						
Duplication	176	77	43.75	13	16.88	.63
Deletion	141	59	41.84	7	11.86	
Duplication/deletion	13	5	38.46	1	20.00	

Abbreviations: CNV, copy number variation; IVF, vitro fertilization; NIPT, noninvasive prenatal testing; PPV, positive predictive value.

^a^ In vitro fertilization.

^b^ Fetal‐free DNA concentration.

^c^ Copy number variation,

* Fisher exact probability method,

## 4 DISCUSSION

In this study, we evaluated the efficiency of NIPT for the identification of fetal autosomal CNVs in pregnant women. Our results showed that 42.73% (141/330) of pregnant women chose further prenatal diagnosis when CNVs were found by NIPT, and 44.95% of pregnant women under 35 years old underwent further prenatal analyses compared with 27.91% of pregnant women over 35 years old. The prenatal diagnosis rate of pregnant women who underwent IVF (72.73%) was higher than that of natural pregnant women (41.69%). Thus, our findings demonstrated that young pregnant women and women with strong reproductive needs were more likely to receive prenatal diagnosis.

Karyotype analysis is considered the “gold standard” for the prenatal diagnosis of chromosomal diseases. However, it is difficult to identify subtle chromosome aberrations less than 5–10 Mb in size. In this study, 14 CNVs detected by NIPT and confirmed by CMA were not found by fetal karyotype analysis; among these, patient 9 had a 9.6‐Mb duplication that was not detected by karyotyping. Thus, NIPT appeared to be superior to karyotyping for smaller CNVs.

Smaller CNVs can be detected by CMA. In this study, 21 fetal samples were found to harbor 23 CNVs by CMA. The sizes of CNVs ranged from 790 kb to 34.7 Mb. Five patients (patients 3, 4, 6, 8, and 10) were found to have pathogenic CNVs, which resulted in the termination of pregnancy to avoid the birth of genetically defective infants. Four likely pathogenic CNVs (patients 1, 7, 9, and 17) were also detected. Two of these CNVs were inherited from their mothers and two were not verified in parents. All four fetuses were born without abnormal appearances and further follow‐up is currently underway. Of the 14 CNVs of uncertain significance and likely benign in 12 fetuses, 11 fetuses were born without abnormalities. Additionally, for one CNV of uncertain significance, after the pregnant mother was informed of the CNV, the patient chose to terminate the pregnancy because there was already a healthy child in their family.

Unfortunately, of the 21 prenatal samples, only seven pregnant couples were willing to undergo peripheral blood CMA to determine whether the fetal CNV was inherited from their parents. Moreover, despite the identification of specific pathogenic CNVs or likely pathogenic CNVs not verified in the parents, most pregnant women chose to continue their pregnancies. Thus, additional work is needed to strengthen education outreach programs and enhance the awareness of genetic diseases in pregnant women.

Noninvasive prenatal testing, which can be used to detect fetal aneuploidy with low coverage, has the potential to detect fetal CNVs, but with false‐positive and false‐negative results (Martin et al., 2018; Sahoo et al., 2016). In our study, the false‐positive rate of CNVs detected by NIPT was 85.1% and the total PPV was 14.89%. Because the fetal‐free DNA concentration and uniquely mapped reads number directly affect the detection efficiency of NIPT (Benn, 2014; Zhao et al., 2015), we used 10% as the critical value group to determine the PPV of CNVs detected by NIPT in different fetal‐free DNA concentration groups and found no significant difference. We used 10 M as the critical value to determine the PPV of CNVs detected by NIPT in a different effective reads array and found no significant difference between the two groups. In addition, the PPVs of the low age group, natural pregnancy group, and duplication group were higher than those of the old age group, IVF group, and deletion group, although the differences were not statistically significant. However, interestingly, we found that the PPV of CNVs less than 10 Mb was significantly higher than that of CNVs greater than 10 Mb detected by NIPT; this finding may be related to the low‐depth sequencing of NIPT and limited data volume.

In summary, in this study, we compared the efficiency of NIPT, karyotype analysis, and CMA in CNV analysis. The results showed that the accuracy of NIPT for autosomal CNV detection in low‐coverage whole‐genome sequencing needs to be improved and the detection of CNVs in small segments by chromosome karyotype analysis also had technical limitations. Based on our current findings, CMA is still the most effective method for CNV detection; however, further analyses are needed to confirm our results. Therefore, chromosome microdeletion/microduplication detected by NIPT should be confirmed by ultrasonic results, karyotype analysis, and CMA to determine the fetal survival. Additionally, in order to obtain better statistical reliability, more studies with greater numbers of specimens are required.

## CONFLICT OF INTEREST

The authors declare that there are no conflicts of interest that could be perceived as prejudicial to the impartiality of the reported research.

## AUTHOR CONTRIBUTION

Yuanyuan Pei and Fengxiang Wei conceived the study, Yuanyuan Pei and Liang Hu analyzed the data and wrote the manuscript, Jinxing Liu, Lijuan Wen, Xiaojin Luo, and Jian Lu conducted the experiments, all authors read and approved the final version.

## Data Availability

All data involved in this manuscript are available.
